# Let There Be Heat: Silica-Coated Gold Nanoparticles as Photothermal Reactors for Chemical Synthesis

**DOI:** 10.1021/acs.accounts.5c00072

**Published:** 2025-04-22

**Authors:** Aritra Biswas, Nir Lemcoff, Yossi Weizmann

**Affiliations:** † Department of Chemistry, Ben-Gurion University of the Negev, Beer-Sheva 84105, Israel; ‡ Ilse Katz Institute for Nanotechnology Science, Ben-Gurion University of the Negev, Beer-Sheva 84105, Israel; § Goldman Sonnenfeldt School of Sustainability and Climate Change, Ben-Gurion University of the Negev, Beer-Sheva 84105, Israel

## Abstract

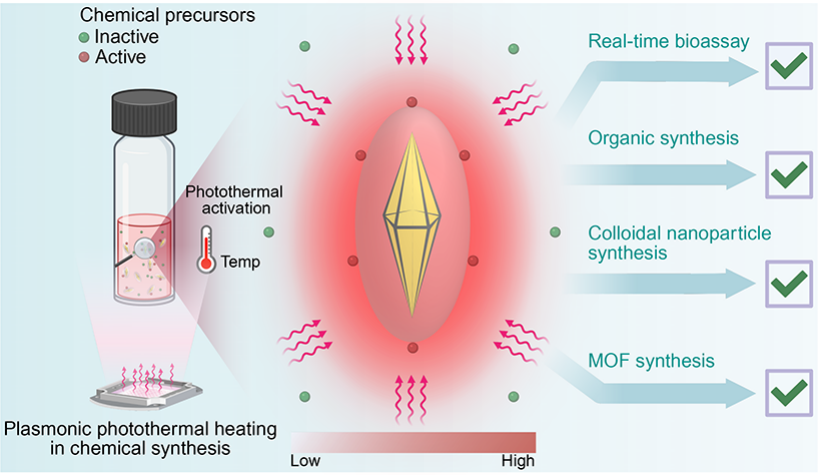

The heating of matter upon interacting with light is a fundamental process ubiquitous in the natural world. With the rise of nanotechnology over the past decades, a variety of nanomaterials capable of converting light into heat have been discovered and their physicochemical properties investigated. Perhaps the most exotic is the photothermal heating of metallic nanocrystals via surface plasmons. Here an incoming electromagnetic wave triggers the oscillation of the nanoparticle’s electron cloud. When in resonance, this generates an enormous increase to the absorption coefficient, enabling more energy to dissipate as heat. The plasmonic phenomenon has an incredibly diverse range of functions, from the vibrant coloration of medieval stained-glass windows to the localization and enhancement of light at the nanoscale level. Plasmonic heating or thermoplasmonics is a relatively new addition that has gained popularity mainly through applications in therapeutics and biotechnology. With this Account, we aim to put a spotlight on the use of thermoplasmonics to drive chemical synthesis, a rapidly expanding area of research with immense potential.

Throughout the long tradition of chemical synthesis, chemists have rarely deviated from the typical oven or hot plate to set and maintain a homogeneous temperature within the reaction vessel. In contrast, the use of thermoplasmonic nanomaterials can introduce heterogeneity to the heating profile of a reaction by forming steep temperature gradients near the surface of nanoparticles. Additionally, photothermal conversion enables heat activated processes to benefit from the advantages of light initiation, e.g., contactless activation and spatial control. Thus, thermoplasmonics offers an attractive alternative to the long-standing norm.

Several early studies demonstrated the power of this method, taking advantage of the localized heating to carry out reactions with minimal change to the bulk temperature of the surrounding medium. However, tapping into this potential can be very challenging as colloidal solutions tend to aggregate even with small changes to the environment. Different strategies have been utilized to overcome this obstacle, for example embedding particles into glass or other heterogeneous substrates. Our group has experimented with coating gold nanostructures with a silica shell. This ensures the structural and colloidal stability that is critical for thermoplasmonic chemistry. Recently, we applied this methodology to advance olefin metathesis, the synthesis of iron oxide (IO), palladium (Pd) and silver (Ag) nanoparticles, and the formation of various metal–organic frameworks (MOFs). In addition, highly stable hybrid materials could be isolated as composites of plasmonic particles with polymers, MOFs, and other nanostructures. The large variety of reaction conditions and the different precursors, additives, and catalysts that our method proved to be compatible with highlight the versatility that silica encapsulation provides. The unique properties of plasmonic heating coupled with the added stability can open a wide range of opportunities for more efficient reactions and altogether new reactivity along with the formation of novel composite materials.

## Key References

- LeeJ.-H.; CheglakovZ.; YiJ.; CroninT. M.; GibsonK. J.; TianB.; WeizmannY.Plasmonic Photothermal Gold Bipyramid Nanoreactors for Ultrafast Real-Time Bioassays. J. Am. Chem. Soc.2017, 139 ( (24), ), 8054–805728457135 10.1021/jacs.7b01779.^1^*This strategy demonstrates ultrafast, low-energy, quantitative real-time PCR with monodisperse PEG-Si-AuBPs as plasmonic photothermal nanoreactors with readily available LEDs. The robust AuBP structure within the encapsulation enhances colloidal stability, enabling a highly efficient process*.
- LemcoffN.; NechmadN. B.; EivgiO.; YehezkelE.; ShelonchikO.; PhatakeR. S.; YesodiD.; VaismanA.; BiswasA.; LemcoffN. G.; WeizmannY.Plasmonic Visible–near Infrared Photothermal Activation of Olefin Metathesis Enabling Photoresponsive Materials. Nat. Chem.2023, 15 ( (4), ), 475–48236702882 10.1038/s41557-022-01124-7.^2^*Using gold nanoparticles’ plasmonic photothermal response to activate latent Ru-based catalysts enabled light-induced olefin metathesis across visible and infrared regions. This approach improved catalyst activation and reaction efficiency, forming polymer–nanoparticle composites with enhanced mechanical properties*.
- BiswasA.; LemcoffN.; ShelonchikO.; YesodiD.; YehezkelE.; FinestoneE. Y.; UpcherA.; WeizmannY.Photothermally Heated Colloidal Synthesis of Nanoparticles Driven by Silica-Encapsulated Plasmonic Heat Sources. Nat. Commun.2023, 14 ( (1), ), 635537816769 10.1038/s41467-023-42167-9PMC10564728.^3^*The thermoplasmonic capability of Au bipyramids was harnessed to initiate the growth of nanoparticles and control the formation of intricate assemblies. This method enabled working with low reaction temperatures and provided higher yields*.
- ShelonchikO.; LemcoffN.; ShimoniR.; BiswasA.; YehezkelE.; YesodiD.; HodI.; WeizmannY.Light-Induced MOF Synthesis Enabling Composite Photothermal Materials. Nat. Commun.2024, 15 ( (1), ), 115438326307 10.1038/s41467-024-45333-9PMC10850081.^4^*The study led to a robust, versatile, and rapid light-induced MOF synthesis using photothermal materials, providing an alternative to energy-intensive solvothermal methods and enabling composite formation for photothermal desorption, and MOF activation*.

## 1 Introduction

The heating of materials upon exposure to light is a process of fundamental importance, most familiar to us through the feel of sunshine on a sunny day. Over the past several decades, advances in nanotechnology have enabled the discovery and development of a wide range of nanomaterials that show an exceptional capability for this light-to-heat conversion.^5^ Photothermal nanoconverters are often classified into three main categories by the physical mechanism of conversion: thermal vibrations, nonradiative relaxation and surface plasmon resonance. Polymers and carbon-based materials (graphene, carbon dots, etc.) absorb photons through the excitation of large, conjugated pi systems that can relax through thermal vibrations generating heat.^6,7^ The second category includes semiconductor nanoparticles (NPs) that can be excited and subsequently undergo nonradiative relaxation, again transforming photons to heat.^8^ Finally, metallic nanostructures particularly of noble metals can have huge absorption cross sections and a potent photothermal response via the localized surface plasmon resonance (LSPR) effect.^9,10^ Each class of materials offers different advantages, such as the low cost of carbon-based materials, the catalytic nature of semiconductor NPs and the tunability of the activation wavelength in metallic NPs, utilizing them to their best effect is an ongoing effort.^5^

Plasmonic materials came to prominence not for their use as nano heaters, but for their ability to concentrate light into extremely small nanoscale pockets.^11^ Initially, the Joule heating resulting from collective oscillations of the plasmonic NP’s electron cloud was thought of as an unwanted byproduct. At a certain frequency the oscillating cloud reaches a state of resonance known as LSPR leading to a great increase in the absorption and thus in the subsequent heating. The large photothermal conversion concentrated into the dimensions of a nanostructure can create steep temperature gradients between the NP surface and its surroundings.^10,12,13^ This unique environment is characterized by localized thermal effects and altered physicochemical properties. The realization that the photothermal side effect could play a central role came around the turn of the century, with the publication of two pioneering works. The first, published in 2002, introduced the concept of plasmonic photothermal imaging^14^ while the second, coming shortly after, showed targeting of cancer tumors by plasmonic hyperthermia.^15^ In both studies the authors made use of the remote activation and spatial resolution provided by light induced heating, features that are absent from conventional heating methods. Moreover, photothermal imaging depends on the formation of nanoscale hotspots around the plasmonic photothermal agents to determine their position, a phenomenon distinctive to photothermal nanomaterials.

In the following decades the interest in plasmonic heating has steadily grown leading to the development of diverse applications.^16^ Sterilization of surfaces,^17−19^ the release of cargoes such as small molecule drugs or genes at specific locations^20,21^ and seawater desalination^22^ are some examples where thermoplasmonics were utilized. In parallel a theoretical framework has been established, aimed to produce predictive tools and thus improve the understanding and design of a given system.^9,12,13,23^ This together with techniques in nanothermometry^24^ have enabled some insight into the temperatures at the surface of the photothermal agents and the gradient going into the surrounding medium. Nonetheless, there are still debates as to the true nature of these localized hotspots mainly due to the difficulty in gathering reliable temperature measurements at the nanoscale. Harnessing photothermal heat to advance chemical reactions is an emerging field in this landscape with huge potential.^25−27^ However, until recently, limited attention has gone into it, with most research staying within the biomedical realm,^28,29^ hardly deviating from the outline set by the seminal works described.^14,15^ Thus, thermoplasmonics offers many opportunities for innovative research especially when looking beyond the aqueous environments and low temperatures of a biochemical setting.

Gold nanoparticles (AuNPs) are by far the most popular choice when searching for a potential thermoplasmonic material. The relatively simple synthesis of different nanostructures enables control over the LSPR wavelength all through the visible and near-infrared (NIR) regions.^30^ Gold is also renowned for being relatively inert providing chemical stability and biocompatibility, considerably expanding the scope of applications.^28,31^ Furthermore, noble metals show exceptional photothermal properties with anisotropic structures such as nanorods or bipyramids reaching extremely high conversion efficiencies and surface temperatures.^32^ These make AuNPs ideal candidates for many thermoplasmonic applications, however other nanomaterials such as silver, copper and titanium nitride have been utilized as well.^33,34^

In this Account we aim to provide an overview of the progress made utilizing plasmonic heating to drive chemical synthesis and introduce the versatile approach of encapsulating gold nanoparticles in silica. We start by describing the project that led us onto this path and giving our assessment of the fundamental advantages of photothermal sources encapsulated in silica. Next, the Account is divided into three sections briefly describing the state-of-the-art in the application of thermoplasmonics in organic, nanocrystal and MOF synthesis. Finally, an outlook on the future of this emerging field is given including potential pathways for development.

## 2 How It Started

**Figure 1:**
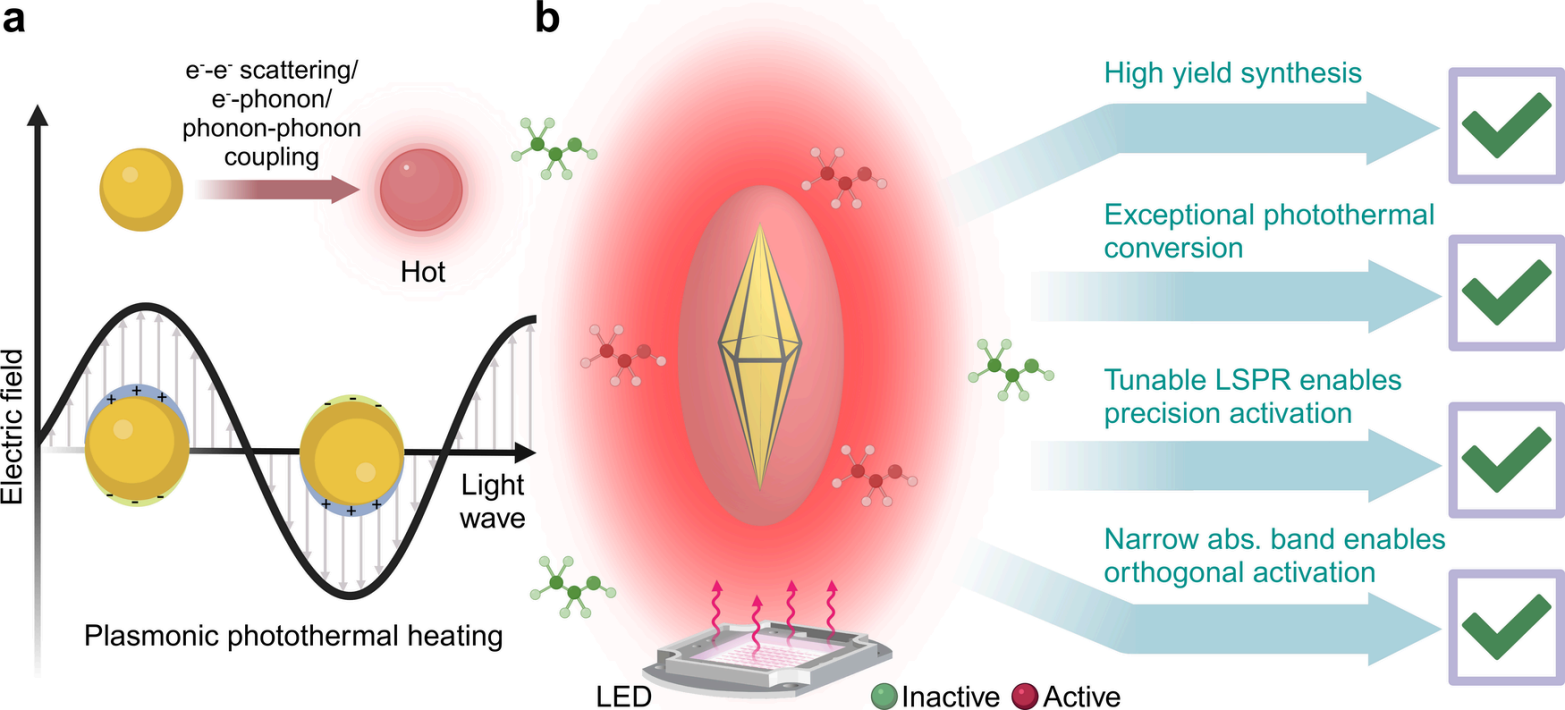
(a) Illustration of localized surface plasmon resonance in gold nanoparticles, demonstrating the way oscillations of the electron cloud occur upon interaction with an incident electromagnetic wave resulting in heat generation. (b) Illustration highlighting the unique properties of gold bipyramids making them ideal candidates for thermoplasmonic synthesis.

Our group’s first experience with thermoplasmonics came almost a decade ago through our interest in nucleic acid amplification techniques.^1^ We had the idea to utilize photothermal conversion to rapidly achieve the temperature cycles necessary to perform a polymerase chain reaction (PCR). This powerful amplification method is normally carried out with heavy heating blocks controlled by expensive Peltier devices and has no point-of-care alternative. Thus, we successfully developed a custom device based on gold nanobipyramids (AuBPs) with LSPR bands at around 850 nm capable of ultrafast real-time quantitative PCR and isothermal amplification assays. Unbeknownst to us then, the choice to work with AuBPs that absorb in the NIR would follow us throughout our journey utilizing thermoplasmonics (Figure 1a). When starting this project, the relatively narrow LSPR bands that could easily be pushed to the NIR by controlling AuBP size were our main consideration. These features ensured the photothermal NPs could fulfill their role without photobleaching the standard fluorescent dyes needed for real time assays. Another challenge was maintaining the structural and colloidal stability of AuBPs under the harsh conditions typical to PCR, including temperatures above 95 °C and high ionic strength. Here silica encapsulation served as an ideal fix, stabilizing the bipyramidal structure and completely preventing aggregation. Additionally, PEGylation of the silica shell could be achieved by a simple silanization procedure with methoxy-poly(ethylene-glycol)-silane, effectively preventing any nonspecific binding of DNA or other biomolecules and adding to the stability of the shell, which may be prone to degradation in certain aqueous conditions.^35^ Thus, the nanoparticles become inert in the solution, efficiently heating the PCR without interfering, eliminating the need for an external heating source.

Overcoming the obstacles in the detailed project and achieving highly efficient light induced heating with a simple custom-built setup motivated us to look further into thermoplasmonics (Figure 1b). At the time few studies had utilized elevated temperatures and explored different solvents beyond an aqueous medium. During our work we realized our method was not limited by the stability of the photothermal agent and could be expanded beyond the biological environment of popular applications such as photothermal therapy.

## 3 Advantages of Silica Encapsulation

In retrospect the encapsulation of AuBPs with silica served as a key that opened for us a wide range of possibilities for thermoplasmonic chemistry. This simple technique was first introduced for AuNPs in 1996 by Liz-Marzan et al., who utilized an amino functionalized silane as a primer from which a silica layer could grow.^36^ Since then, this technology has rapidly evolved with different strategies enabling control over porosity, thickness and functionality including selective deposition in anisotropic structures.^37,38^ In our work, we utilize a two-step protocol initially covering the AuNP with a thick layer of mesoporous silica and subsequently forming a thin dense layer by applying the Stöber method. Although silica NPs are not impervious to aggregation or degradation, especially in aqueous and saline conditions,^39^ this protocol has afforded remarkably stable NPs under the extremely harsh conditions described in the following sections of this Account.

**Figure 2:**
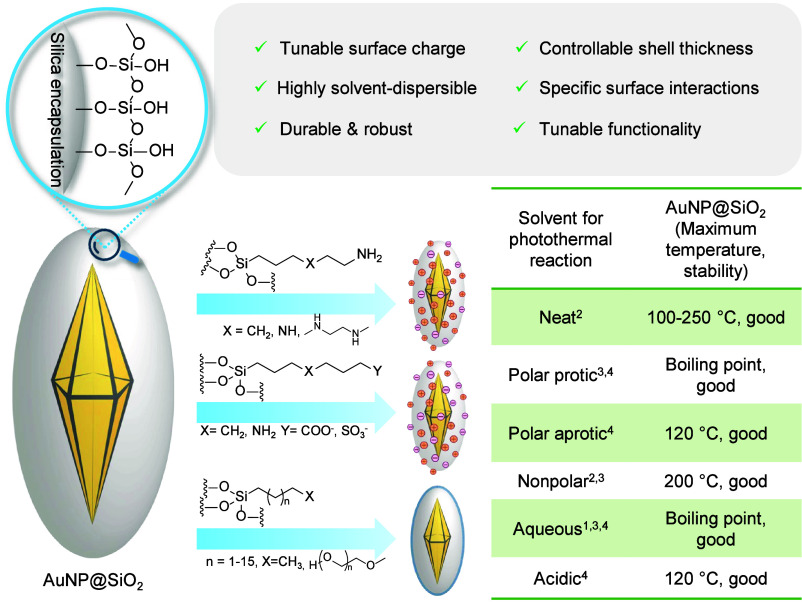
Impact of silica encapsulation on nanoparticle stabilization and the introduction of surface functionality for thermoplasmonic chemical reactions under versatile conditions, including high temperatures, extreme pH, and oxidative environments, enabling efficient synthesis. A bipyramidal structure is illustrated; however, these principles apply to any Au geometry that can be encapsulated.

As we view it, there are three critical advantages brought on by the presence of a silica shell enabling the exceptional versatility of the described methodology (Figure 2):

I. **Structural stability**. The geometry of plasmonic nanoparticles has a direct influence on their optical properties and thus on their photothermal conversion. Nonspherical structures tend to undergo deformation when exposed even to mild oxidizing conditions including exposure to air, leading to a blue shift in the LSPR. This effect is enhanced at elevated temperatures where melting can also occur. When utilizing a laser or LED with a narrow wavelength distribution, minor changes in structure lead to a detrimental loss in photothermal conversion, preventing the use of complex structures in any reaction that is exposed to the atmosphere. Silica encapsulation prevents the degradation of the structure under extremely harsh conditions. In our research we have been able to carry out photothermal reactions at above 200 °C and hold this temperature for several hours with minimal etching of AuBP tips.^2,3^
II. **Colloidal stability**. Perhaps the greatest challenge of employing nanoparticles as photothermal heat sources is dispersing them in a solution and maintaining their stability throughout the reaction. This problem is not exclusive to thermoplasmonic chemistry, nanoparticles are popular catalysts for a variety of reactions. The traditional strategies to overcome this can be roughly divided between the selection of proper ligands and the use of a heterogeneous system in which the NP catalyst is usually attached to a solid phase. In the context of thermoplasmonics the scope of possible ligands is very limited due to the intense temperatures at the surface of the photothermal source and the relatively weak bonds that connect them to NPs. Thus, most studies demonstrating a thermoplasmonic source utilize heterogeneous systems. For example, gold spheres embedded into glass or polymeric substrates^40,41^ or PdNPs within large MOF crystals.^42^ These and similar studies are important, however a homogeneous methodology can provide improved efficiency and greater control. Encapsulation with silica allows for exceptional colloidal stability in a wide range of solvents with very different properties. In our work we demonstrate reactions in hydrophobic solvents such as toluene and octadecene in addition to highly polar solvents like dimethylformamide and ethanol. Furthermore, the silica shell can tolerate highly acidic conditions and a wide range of functional groups. This versatility opens the possibility to explore an enormous scope of photothermal reactions without the need to adjust conditions for a stable dispersion.
III. **Surface functionalization**. Silica surfaces can be modified by a relatively simple procedure with a variety of readily available organosilanes. Essentially any type of functionality can be added to the thermoplasmonic NP in this manner. On a conceptual level it is similar to the chemistry developed by attaching thiolated ligands to the surface of AuNPs,^43,44^ with the advantage that the Si–C or Si–O bonds provide a stronger link than the Au–S bond. Thus, the surface chemistry of the silica encapsulated AuNPs (AuNP@SiO_2_) can be tuned to best suit the system of interest. In our work, we utilized this feature to improve the solubility of AuNPs in apolar conditions by attaching alkyl silanes. Additionally, we varied the surface chemistry to test whether the surface played a role in the reaction mechanism. Looking forward, the modular nature of this system could become a platform for a variety of applications. Thinking of thermoplasmonic chemistry we envision this becoming a tool to increase the effective concentration of reactants in the vicinity of the photothermal source where the temperature is high. A concept that can be seen as the inverse of the PEGylation we utilized to deter binding of biomolecules to the polar silica surface in our project on photothermal PCR. In addition, methodologies for selective deposition of silica could be harnessed to introduce different environments via dual functionalization.^45^

## 4 Plasmonic Heating in Organic Synthesis

**Figure 3:**
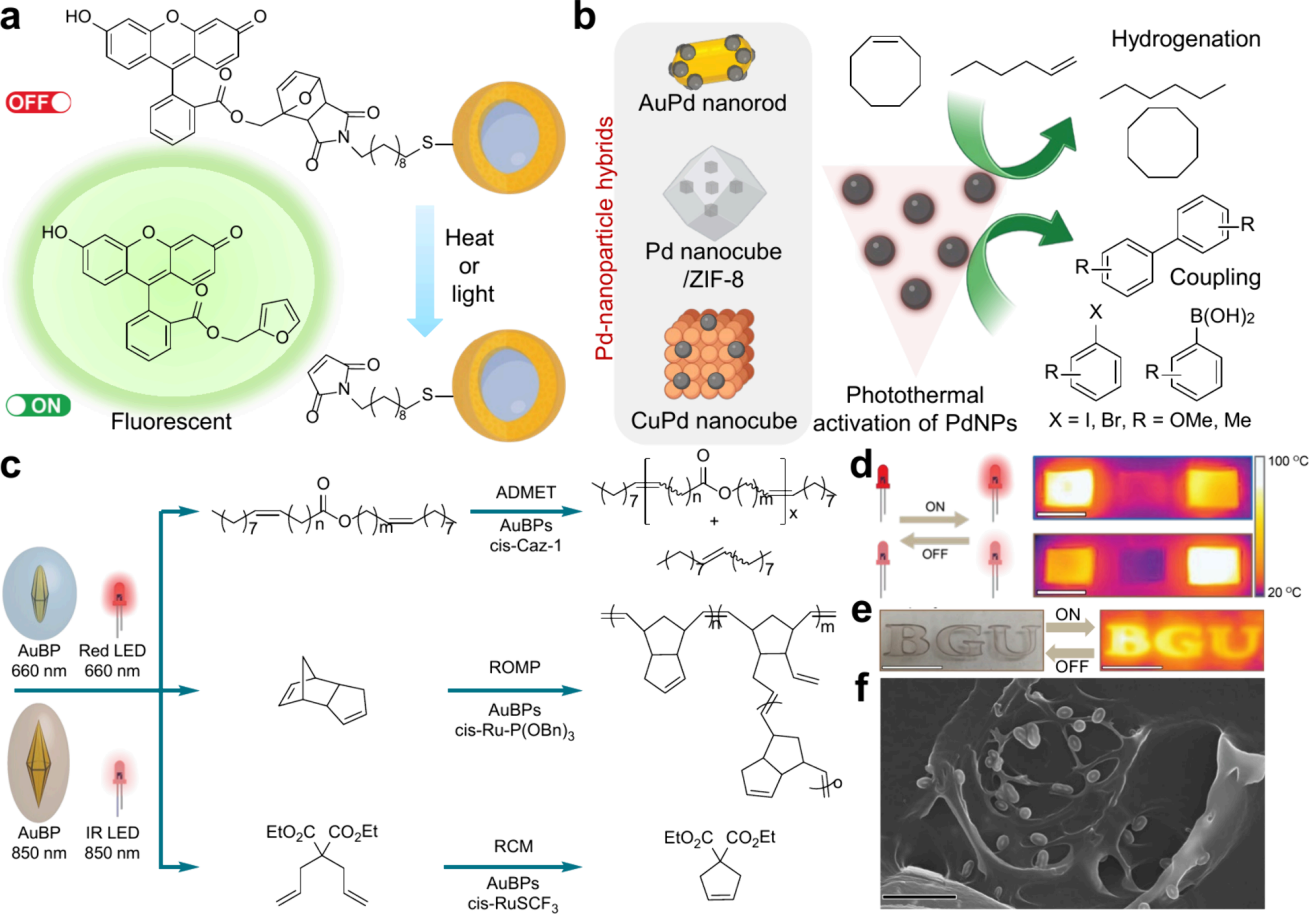
(a) Photothermal activation of the retro-Diels–Alder reaction enables the controlled release of fluorescein dye molecules from the surface of silica-gold core–shell and spherical gold nanoparticles. (b) Photothermal activation of PdNPs for hydrogenations and carbon cross-couplings, with hybridization/encapsulation providing stability and enhanced photothermal properties. (c) Scheme showing plasmonic visible–near-infrared photothermal activation enabling olefin metathesis reactions. (d) Photothermally prepared p-DCPD rectangles: PPC_660_ (left), p-DCPD (No AuBPs, center), and PPC_850_ (right), alongside thermal camera images during irradiation with 660 nm (top) and 850 nm (bottom) light. On the far right is a temperature scale. Scale bars: 10 mm. (e) High-resolution printing of DCPD/*cis*-Ru-P(OBn)_3_/AuBP_850_ formulation with a thermal camera image during irradiation with 850 nm light. Scale bars: 10 mm. (f) SEM image of plasmon-cured PPC_850_, with high glass transition temperatures and tensile strength. Scale bar: 1 μm. Images are reproduced with permission from ref (2). Copyright 2023 Springer Nature.

Organic chemistry is a pillar of modern chemistry and a discipline that has changed society as we know it. The long and impressive tradition of reactions and synthesis procedures, many of which necessitate thermal initiation, almost exclusively relies on bulk heating of reactant solutions. Thus, photothermal organic reactions, in particular with thermoplasmonic nanoparticles enabling localized heating, can provide a powerful alternative to the long-standing paradigm. An early report by the Branda group hints at the potential of this method by demonstrating a retro-Diels–Alder reaction without any noticeable change in the bulk temperature. In their report, a small molecule connected by a thiolated linker to the AuNP surface is detached by decomposition of the linker into a maleimide and a furan. A drawback that was pointed out by the authors was the partial dissociation of thiols from the surface resulting in reactive species problematic for the biological applications they imagined (Figure 3a).^46^ A later approach, aimed more for application in organic synthesis, is the photothermal activation of PdNPs. The combination of both photothermal conversion in the visible region and catalytic activity for hydrogenations and cross couplings situates PdNPs as attractive candidates.^47,48^ To compensate for the relatively poor photothermal conversion of Pd, different groups used hybrid structures with Au or Cu, allowing for an increased photothermal response while still benefiting from palladium’s catalytic properties (Figure 3b).^42,49,50^ Recently, Stache and co-workers have excellently reviewed the progress achieved within this emerging field, telling this story in more detail.^51^

Based on our results from the development of the photothermal PCR, we reasoned that the impressive stability of the AuNP@SiO_2_ could be leveraged to introduce an added level of complexity to the relatively narrow scope of photothermal organic reactions. To this end, we collaborated with the Lemcoff group and developed a thermoplasmonic method to advance olefin metathesis reactions.^2^ Our strategy was to activate thermally latent, Ru-based olefin metathesis catalysts with the photothermal response of SiO_2_ encapsulated AuBPs and gold nanorods (AuNRs). By tuning the size of the AuNPs control over the photothermal activation wavelength was gained, leading to unprecedented reactivity in far-red and NIR wavelengths. Additionally, photothermal reactions showed improved conversions at identical bulk temperatures including particularly difficult reactions such as the formation of tetra-substituted olefins. After an in-depth analysis we concluded that this was the result of an increase in catalyst activity which we attributed to elevated local temperatures around the AuNPs (Figure 3c).

Thermoplasmonic reactors as shown in the aforementioned studies can also be recycled by filtration or centrifugation. Recycling coupled with reducing the temperature of important reactions hints at the potential of thermoplasmonics to impact green chemistry. In addition, decreasing reaction temperatures could also open the possibility to test reactions in solvents with lower boiling points, perhaps unlocking new reactivity and simplifying workup steps.

### 4.1 Photothermal Polymerizations

In our work on olefin metathesis we demonstrated that the thermoplasmonic method could be applied for ring opening polymerization (ROMP) of dicyclopentadiene and acyclic diene polymerization (ADMET) of jojoba oil (Figure 3c). Here too we observed a more efficient reaction for both materials, leading to polymers with improved thermomechanical properties. The Lear group working on the formation of polyurethanes by irradiating gold nanospheres with a 532 nm laser, also reported increased cross-linking and amazingly a billion-fold polymerization rate improvement.^52,53^ Furthermore, the increased degree of polymerization was also demonstrated for methacrylate and epoxy resins working with AgNPs.^54^ Thus, thermoplasmonic polymerizations can serve as an excellent technique to improve the properties of polymeric materials.

A consequence of utilizing the photothermal approach is the entrapment of the plasmonic particles within the polymer matrix once the reaction is complete. In our project on olefin metathesis we demonstrated that the hybrid material took on the photothermal properties of the AuBP@SiO_2_ resulting in what is known as a plasmonic polymer composite (PPC).^55^ The photothermal heating of the composite proved to be incredibly stable and was activated only by the LSPR wavelength, again enabling tunability (Figure 3d–f). Another advantage of transforming a process to light activated is the spatial resolution that can be obtained. In the context of polymerization this has an added value as it opens a path to 3D printing applications. Fedoruk et al. showed an example of this by optically trapping a gold nanosphere with a laser that corresponded to its LSPR wavelength. Thus, the authors could “print” nanoscale objects of polydimethylsiloxane.^56^

## 5 Thermoplasmonic Synthesis of Inorganic NPs

**Figure 4:**
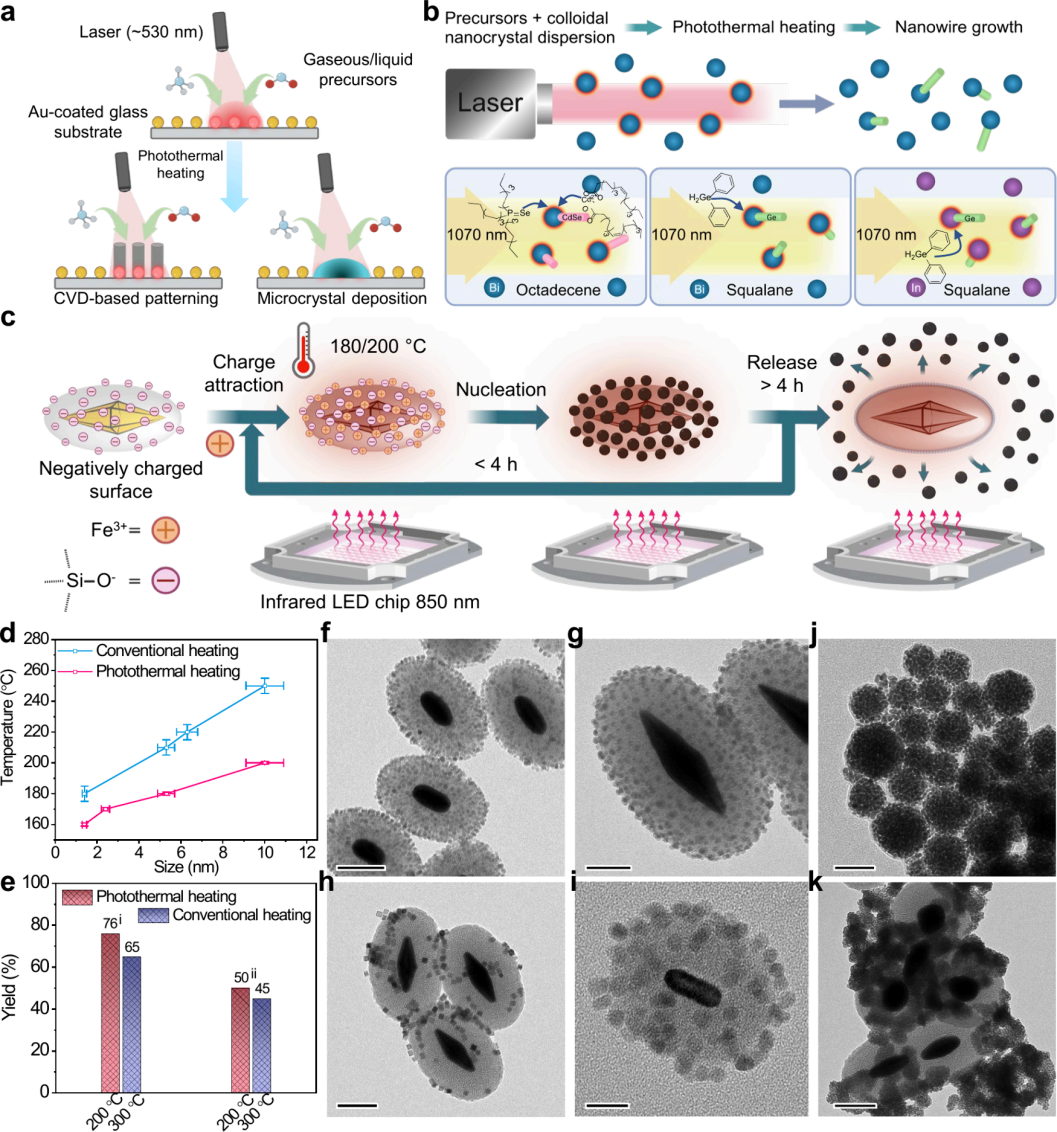
(a) Plasmon-assisted local temperature control using Au-coated or Au-lined glass/quartz substrates for catalysis, nanoscale patterning, and microcrystal growth through CVD or solvothermal reactions. (b) Depiction of a contact-free, solution-phase, photothermal growth process for nanowires (ionic/covalent) conducted on the benchtop in a quartz cuvette utilizing various metal nanocrystal seeds under 1070 nm laser excitation. (c–e) Photothermal heating enables the colloidal synthesis of IONPs using AuBP@SiO_2_ or AuNR@SiO_2_ under 660 or 850 nm LED irradiation. This process leverages localized nucleation and growth, followed by nanoparticle release into the solution. The approach facilitates large-scale production of nanoparticles, including a variety of sizes, shapes, and core–satellite structures, with high yields and at reduced temperatures. (d) Comparison of photothermally synthesized nanoparticles with those produced via conventional heating for a particular size: ∼5 nm particles at 180 °C and ∼10 nm particles at 200 °C (with standard deviation shown as error bars). (e) IONP synthesis results include: (i) ∼2–4 nm particles and (ii) ∼15 nm nanoplates synthesized at 200–300 °C. (f–k) Generality of this concept was further explored in the synthesis of various other nanoparticle morphologies and their core–satellite structure/assembly, including Ag and Pd: (f) AuBP@IONP, (g) AuBP@AgNP, (h) AuBP@Pd-nanocube, (i) AuNR@IONP, (j) Pd assemblies, and (k) AuBP@Pd. Scale bars: g, j: 50 nm; f, h, k: 100 nm; i: 20 nm. Images are reproduced from ref (3) (available under a CC-BY license. Copyright 2023, Aritra Biswas et al., Springer Nature) and ref (61) (Copyright 2021 American Chemical Society).

The fabrication of nanomaterials through the localized heating of plasmonic NPs has also attracted considerable interest. First reports precede photothermal organic synthesis by several years, however, the accumulative effort is less comprehensive with reports being more sporadic. In 2006, a pioneering study by Boyd et al. presented a system where chemical vapor deposition onto a glass surface embedded with AuNPs enabled the patterning of PbO and TiO_2_ by locally heating the AuNPs with a laser (Figure 4a).^57^ Normally the synthesis of metal oxides and other semiconductor nanoparticles proceeds via thermal decomposition of metal complexes containing the relevant atoms. Transforming the decomposition to a light-induced process allows exceptional spatial control of these highly applicable materials. Following studies developed this idea to include more materials and different conditions.^58^ For example, Brongersma et al. expanded the use of a gas–solid interface to the thermoplasmonic lithography of Si nanowires and carbon nanotubes (Figure 4a).^59^ A more recent work by the Baffou group utilized AuNPs on a glass substrate in an aqueous environment to advance the hydrothermal synthesis of indium hydroxide microcrystals (Figure 4a). Notably, here the authors could estimate the temperature gradient around the NP by analyzing the spatial distribution of the product, they concluded that a temperature exceeding 200 °C was reached.^60^ As mentioned previously, attempting to translate the photothermal formation of nanocrystals to homogeneous dispersions results in major stability issues, especially when the decomposition of precursors necessitates elevated temperatures. Recently Pandres and co-workers demonstrated a first example of this reaction, in which they utilize bismuth and indium nanoparticles to grow CdSe and Ge nanowires (Figure 4b). They utilize a high-powered laser to rapidly elevate the local temperature around the metallic NPs inducing nanowire formation from their surface.^61^

In our work on the polymerization of jojoba we found that the AuNP@SiO_2_ could elevate the solution temperature above 200 °C and maintain it for several hours. Thus, we were tempted to test our method in highly demanding synthesis procedures requiring harsh conditions. The formation of γ-Fe_2_O_3_ nanoparticles (IONPs) is such an example and provides a product with outstanding magnetic properties used in a range of applications. In this study, we consistently produced solution temperatures of around 200 °C with silica encapsulated AuNRs and two sizes of AuBPs (Figure 4c–e).^3^ The formation of three different morphologies of IONPs was demonstrated with higher yields at considerably lower temperatures than conventional heating methods. Interestingly, an analysis of the photothermal sources showed that at the initial stages of the reaction, the silica shell was densely decorated with IONPs and that a gradual dissociation was occurring up to a point where the silica was completely bare by the end of the reaction. We hypothesized that a gradual reaction between the silica and the oleic acid ligands occurred changing the surface chemistry leading to the dissociation of IONPs. This was confirmed by modifying the silica cap with a hydrophobic alkylsilane to mimic the surface at the end of the process resulting in bare silica throughout the reaction. Building on these results, the synthesis of Pd and Ag NPs including cubes and wires was also accomplished. Overall, five different solvents (toluene, ethanol, ethylene glycol, oleylamine and 1-octadecene) with a range of different ligands and precursors were utilized highlighting the incredible versatility of AuNP@SiO_2_.

### 5.1 Formation of Superstructures

Notably, during the synthesis of both Pd and Ag NPs, nucleation predominantly occurred on the silica coating of the thermoplasmonic NPs. In all three examples, IO, Pd and Ag, stable hybrid structures could be isolated and showed properties of both the core gold and the peripheral particles (Figure 4f–i). A similar idea was also proposed in the work by Panders et al.^61^ where they suggest utilizing their method to introduce dopants into the “matchstick” particles they produced. Another interesting phenomenon we observed during the formation of PdNPs, was the photothermal contribution of the just synthesized structures. Pd can also act as a photothermal agent thus by irradiating at a suitable wavelength it can *in situ* become a photothermal source in the reaction, adding another layer of complexity to the photothermal process (Figure 4j,k). Under suitable conditions this resulted in large assemblies comprised of numerous small spherical PdNPs. Moreover, by further optimizing the conditions, the PdNP assemblies formed on the original AuNP@SiO_2_ photothermal sources. Normally, structures of this kind require careful design of multiple steps including different additives and ligands aimed to keep the particles in a delicate balance between completely dispersing and aggregating. These unique examples of highly sophisticated superstructures may be the tip of the iceberg when it comes to the scope of possibilities opened by thermoplasmonic synthesis.

## 6 Thermoplasmonic Formation of MOFs

**Figure 5:**
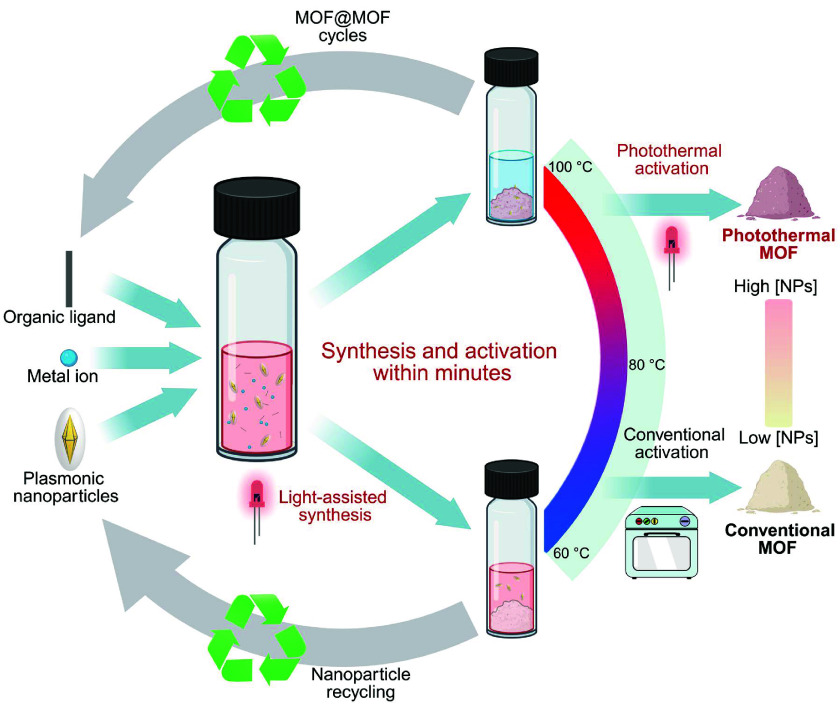
Scheme of light-induced MOF synthesis using silica-encapsulated AuNS/NR/BP. This method was demonstrated to be a robust, versatile, and rapid alternative to conventional solvothermal methods. The AuNPs were embedded into UIO-66 *in situ* through zirconium linkage to the silica shell, creating a photoresponsive composite (AuNP@UIO-66) with retained photothermal properties. Repeated photothermal activation maintained both MOF structure and heat efficiency, enabling ultrafast MOF activation, and photothermal desorption.

The successful syntheses of both organic and inorganic materials pushed us to develop a photothermal alternative to MOFs synthesis (Figure 5). As far as we know our work on the topic was the first of its kind, with other groups taking directions such as microwave heating and sonochemistry to innovate on the traditional solvothermal methods.^62,63^ This study resulted in the photothermal synthesis of four different MOFs (UIO-66, MIL-88A, HKUST-1, and MOF-5) at three different wavelengths (520, 660, and 850 nm) including the use of silica coated gold nanospheres (AuNS), rods and bipyramids. Again, the plasmonic heating yielded rapid reactions, sometimes cutting the reaction from several hours to minutes. Notably, the synthesis of the zirconium based UIO-66 was carried out in a mixture of dimethylformamide and water in highly acidic conditions. Analysis of the photothermally prepared UIO-66 product showed increased surface areas matching reports where crystal defects were intentionally induced to achieve this effect. Put together with more evidence this led us to hypothesize that the rapid formation and highly defected structure were a consequence of localized heating generated by the NPs. Another interesting aspect of this synthesis was the identification of an interaction between the silica shell and the ZrCl_4_ precursor that occurred when conducting the reactions at temperatures above 100 °C. This facilitated the insertion of AuNPs into the MOF matrix resulting in stable AuNP-MOF composites. Thus, the photothermal process provided a light-mediated means to control the formation of these highly functional hybrid materials. Investigating the properties of the composites showed that the photothermal capabilities were intact, elevating the temperature of the dried MOF powder to roughly 250 °C without compromising the unique MOF structure. The possibility to form hybrids with MOF microcrystals completes a series of advanced materials produced via our methodology together with the bulk polymeric composites and the nanoscale superstructures.

## 7 Conclusion and Outlook

The use of plasmonic heating to initiate chemical reactions is an emerging field that has been gaining traction with advancements in the synthesis of plasmonic nanomaterials and our understanding of their properties. An inherent advantage of the photothermal methodology is contactless activation and precise spatial control, unlocking numerous possibilities for applications development. Thermoplasmonic materials also enable the heterogeneous heating discussed above, these hotspots have been shown to introduce unique reactivity through thermal effects without changing the reaction pathway. Our group has contributed to this effort by encapsulating the photothermal agents with silica shells drastically improving their stability and in so greatly expanding the space of possible reactions. The AuNP@SiO_2_ system has served as a modular platform, enabling control over activation wavelength and surface chemistry in a diverse range of environments. This step forward in terms of the method’s versatility is crucial for harnessing the unique properties of thermoplasmonics in an ever-increasing number of synthetic procedures (Table 1).

**Table 1:** Photothermal Applications of AuNP@SiO_2_

Au structure	surface functionality	wavelength (nm)	photothermal temp. (°C)	environment	stability, recyclability	chemical synthesis/application
bipyramid	Si–OH, PEG	850	72–95	aqueous, buffer	good, yes	real-time quantitative PCR^1^
bipyramid, rod	Si–OH	660, 850	80–240	neat, apolar	good, yes	olefin metathesis,^2^ polymerization,^2^ printing^2^
bipyramid, rod	Si–OH, CH_3_, NH_2_	660, 850	70–200	apolar, polar protic, aqueous	good, yes	nanoparticles,^3^ superstructures^3^
bipyramid, rod, sphere	Si–OH	520, 660, 850	60–120	polar protic, aprotic, aqueous, acidic	good, yes	MOFs,^4^ composites,^4^ MOF activation^4^

Nevertheless, as with any emerging technology there are still challenges that remain. Among them is the reliable determination of temperature at the nanoscale, crucial for our understanding of photothermal reactions. Current nanothermometry methods are often indirect, giving rough estimates of the temperature that reactants at the surface may be exposed to.^60,64^ Improving the accuracy of data would enable theoretical models with better predictive abilities, giving a clearer view of the true potential of this methodology. Another more practical problem is the uncertainty over the scalability of thermoplasmonic reactions. When thinking of heating on an industrial scale light-activation poses an obstacle and may be a disadvantage when compared to the well-established conventional methods. Perhaps flow reactors as shown in the work of Pandres et al.^61^ could provide a means of facilitating the transition of photothermal chemistry from lab to industry. Another setback in comparison with conventional heating could be the added cost of introducing a thermoplasmonic agent, here recycling the NPs could greatly reduce the impact.

Thermoplasmonic systems that include a catalyst within the thermal hotspot are especially interesting as this combination can lead to impressive results. Looking forward, integrating the versatility offered by silica coating into this type of system presents an intriguing challenge. One option would be to utilize porous silica or partial encapsulation by selective deposition to allow access to the catalytic surface. However, exposing the plasmonic nanostructures would also render them vulnerable to etching. Utilizing silane chemistry to attach either a homogeneous catalyst or active NPs creating a reactive outer layer may lead to highly potent materials, though the added complexity will result in demanding syntheses. Another interesting prospect is to modify the silica shell to enhance its affinity for the reactants, thereby increasing their effective molarity within the high temperature zone. This could be done by mismatching the properties of the surface and the solvent to an extent that still enables a dispersion but increases the probability of adsorption to the modified silica surface. Lastly, changing the shell material altogether is also possible as shown by the Wang group in their work on titania encapsulations.^65^ Here the versatility of silica modification is traded for a more reactive material according to the requirements of the desired system.

Finally, throughout this Account we have elaborated on the composite materials that the silica coated NPs could form with practically any material that was produced. A full series from the nanoscale superstructures to the microcrystalline MOF hybrids and all the way to the bulk PPCs were demonstrated to be almost inevitable. These highly valuable advanced materials could be utilized in a wide range of applications, e.g., plasmonic MOFs have the potential to realize rapid photoinduced absorption–desorption cycles of energy dense gases or serve to enhance the outstanding inherent catalytic properties of MOFs.^4^ PPCs have also been shown to be highly applicable in 4D printing techniques, shape-morphing devices and robotics, in addition to the exciting idea of utilizing the entrapped particles to induce photothermal depolymerization.^55,66^ Thus, as the pioneers of this field thought to exploit the “unwanted” byproduct we think this could be a major point of focus moving toward the future. This methodology could be leveraged to facilitate access to increasingly complicated materials without the degree of chemical trickery that might be required otherwise. To conclude, the use of plasmonic heating without needing to alter reaction conditions to accommodate a colloidal dispersion opens a sea of opportunities for creative research and innovation that is sure to impact chemical synthesis in the coming years.

## References

[ref1] Lee J.-H.; Cheglakov Z.; Yi J.; Cronin T. M.; Gibson K. J.; Tian B.; Weizmann Y. Plasmonic Photothermal Gold Bipyramid Nanoreactors for Ultrafast Real-Time Bioassays. J. Am. Chem. Soc. 2017, 139 (24), 8054–8057. 10.1021/jacs.7b01779. 28457135

[ref2] Lemcoff N.; Nechmad N. B.; Eivgi O.; Yehezkel E.; Shelonchik O.; Phatake R. S.; Yesodi D.; Vaisman A.; Biswas A.; Lemcoff N. G.; Weizmann Y. Plasmonic Visible–near Infrared Photothermal Activation of Olefin Metathesis Enabling Photoresponsive Materials. Nat. Chem. 2023, 15 (4), 475–482. 10.1038/s41557-022-01124-7. 36702882

[ref3] Biswas A.; Lemcoff N.; Shelonchik O.; Yesodi D.; Yehezkel E.; Finestone E. Y.; Upcher A.; Weizmann Y. Photothermally Heated Colloidal Synthesis of Nanoparticles Driven by Silica-Encapsulated Plasmonic Heat Sources. Nat. Commun. 2023, 14 (1), 6355 10.1038/s41467-023-42167-9. 37816769 PMC10564728

[ref4] Shelonchik O.; Lemcoff N.; Shimoni R.; Biswas A.; Yehezkel E.; Yesodi D.; Hod I.; Weizmann Y. Light-Induced MOF Synthesis Enabling Composite Photothermal Materials. Nat. Commun. 2024, 15 (1), 1154 10.1038/s41467-024-45333-9. 38326307 PMC10850081

[ref5] Cui X.; Ruan Q.; Zhuo X.; Xia X.; Hu J.; Fu R.; Li Y.; Wang J.; Xu H. Photothermal Nanomaterials: A Powerful Light-to-Heat Converter. Chem. Rev. 2023, 123 (11), 6891–6952. 10.1021/acs.chemrev.3c00159. 37133878 PMC10273250

[ref6] Xu D.; Li Z.; Li L.; Wang J. Insights into the Photothermal Conversion of 2D MXene Nanomaterials: Synthesis, Mechanism, and Applications. Adv. Funct. Mater. 2020, 30 (47), 2000712 10.1002/adfm.202000712.

[ref7] Yu C.; Xu L.; Zhang Y.; Timashev P. S.; Huang Y.; Liang X.-J. Polymer-Based Nanomaterials for Noninvasive Cancer Photothermal Therapy. ACS Appl. Polym. Mater. 2020, 2 (10), 4289–4305. 10.1021/acsapm.0c00704.

[ref8] Hessel C. M.; Pattani V. P.; Rasch M.; Panthani M. G.; Koo B.; Tunnell J. W.; Korgel B. A. Copper Selenide Nanocrystals for Photothermal Therapy. Nano Lett. 2011, 11 (6), 2560–2566. 10.1021/nl201400z. 21553924 PMC3111000

[ref9] Jauffred L.; Samadi A.; Klingberg H.; Bendix P. M.; Oddershede L. B. Plasmonic Heating of Nanostructures. Chem. Rev. 2019, 119 (13), 8087–8130. 10.1021/acs.chemrev.8b00738. 31125213

[ref10] Baffou G.; Quidant R. Thermoplasmonics. In World Scientific Handbook of Metamaterials and Plasmonics; World Scientific Series in Nanoscience and Nanotechnology; World Scientific, 2017; pp 379–407, 10.1142/9789813228726_0010.

[ref11] Schuller J. A.; Barnard E. S.; Cai W.; Jun Y. C.; White J. S.; Brongersma M. L. Plasmonics for Extreme Light Concentration and Manipulation. Nat. Mater. 2010, 9 (3), 193–204. 10.1038/nmat2630. 20168343

[ref12] Baffou G.; Quidant R. Thermo-Plasmonics: Using Metallic Nanostructures as Nano-Sources of Heat. Laser & Photonics Reviews 2013, 7 (2), 171–187. 10.1002/lpor.201200003.

[ref13] Govorov A. O.; Richardson H. H. Generating Heat with Metal Nanoparticles. Nano Today 2007, 2 (1), 30–38. 10.1016/S1748-0132(07)70017-8.

[ref14] Boyer D.; Tamarat P.; Maali A.; Lounis B.; Orrit M. Photothermal Imaging of Nanometer-Sized Metal Particles Among Scatterers. Science 2002, 297 (5584), 1160–1163. 10.1126/science.1073765. 12183624

[ref15] Hirsch L. R.; Stafford R. J.; Bankson J. A.; Sershen S. R.; Rivera B.; Price R. E.; Hazle J. D.; Halas N. J.; West J. L. Nanoshell-Mediated near-Infrared Thermal Therapy of Tumors under Magnetic Resonance Guidance. Proc. Natl. Acad. Sci. U. S. A. 2003, 100 (23), 13549–13554. 10.1073/pnas.2232479100. 14597719 PMC263851

[ref16] Baffou G.; Cichos F.; Quidant R. Applications and Challenges of Thermoplasmonics. Nat. Mater. 2020, 19 (9), 946–958. 10.1038/s41563-020-0740-6. 32807918

[ref17] Neumann O.; Feronti C.; Neumann A. D.; Dong A.; Schell K.; Lu B.; Kim E.; Quinn M.; Thompson S.; Grady N.; Nordlander P.; Oden M.; Halas N. J. Compact Solar Autoclave Based on Steam Generation Using Broadband Light-Harvesting Nanoparticles. Proc. Natl. Acad. Sci. U. S. A. 2013, 110 (29), 11677–11681. 10.1073/pnas.1310131110. 23836642 PMC3718182

[ref18] Reinhard B. M. Plasmonic Enhancement Strategies for Light-Driven Microbe Inactivation. J. Phys. Chem. C 2022, 126 (5), 2325–2335. 10.1021/acs.jpcc.1c09951. PMC9611023 36313122

[ref19] de Miguel I.; Prieto I.; Albornoz A.; Sanz V.; Weis C.; Turon P.; Quidant R. Plasmon-Based Biofilm Inhibition on Surgical Implants. Nano Lett. 2019, 19 (4), 2524–2529. 10.1021/acs.nanolett.9b00187. 30860848

[ref20] Florentsen C. D.; West A.-K. V.; Danielsen H. M. D.; Semsey S.; Bendix P. M.; Oddershede L. B. Quantification of Loading and Laser-Assisted Release of RNA from Single Gold Nanoparticles. Langmuir 2018, 34 (49), 14891–14898. 10.1021/acs.langmuir.8b01831. 30407836

[ref21] Huschka R.; Zuloaga J.; Knight M. W.; Brown L. V.; Nordlander P.; Halas N. J. Light-Induced Release of DNA from Gold Nanoparticles: Nanoshells and Nanorods. J. Am. Chem. Soc. 2011, 133 (31), 12247–12255. 10.1021/ja204578e. 21736347 PMC4108302

[ref22] Zhou L.; Tan Y.; Wang J.; Xu W.; Yuan Y.; Cai W.; Zhu S.; Zhu J. 3D Self-Assembly of Aluminium Nanoparticles for Plasmon-Enhanced Solar Desalination. Nat. Photonics 2016, 10 (6), 393–398. 10.1038/nphoton.2016.75.

[ref23] Baffou G.; Quidant R.; García de Abajo F. J. Nanoscale Control of Optical Heating in Complex Plasmonic Systems. ACS Nano 2010, 4 (2), 709–716. 10.1021/nn901144d. 20055439

[ref24] Quintanilla M.; Liz-Marzán L. M. Guiding Rules for Selecting a Nanothermometer. Nano Today 2018, 19, 126–145. 10.1016/j.nantod.2018.02.012.

[ref25] Baffou G.; Quidant R. Nanoplasmonics for Chemistry. Chem. Soc. Rev. 2014, 43 (11), 3898–3907. 10.1039/c3cs60364d. 24549257

[ref26] Mateo D.; Cerrillo J. L.; Durini S.; Gascon J. Fundamentals and Applications of Photo-Thermal Catalysis. Chem. Soc. Rev. 2021, 50 (3), 2173–2210. 10.1039/D0CS00357C. 33336654

[ref27] Song C.; Wang Z.; Yin Z.; Xiao D.; Ma D. Principles and Applications of Photothermal Catalysis. Chem. Catalysis 2022, 2 (1), 52–83. 10.1016/j.checat.2021.10.005.

[ref28] Ruhoff V. T.; Arastoo M. R.; Moreno-Pescador G.; Bendix P. M. Biological Applications of Thermoplasmonics. Nano Lett. 2024, 24 (3), 777–789. 10.1021/acs.nanolett.3c03548. 38183300 PMC10811673

[ref29] Kim M.; Lee J.-H.; Nam J.-M. Plasmonic Photothermal Nanoparticles for Biomedical Applications. Advanced Science 2019, 6 (17), 1900471 10.1002/advs.201900471. 31508273 PMC6724476

[ref30] Vinnacombe-Willson G. A.; Conti Y.; Stefancu A.; Weiss P. S.; Cortés E.; Scarabelli L. Direct Bottom-Up In Situ Growth: A Paradigm Shift for Studies in Wet-Chemical Synthesis of Gold Nanoparticles. Chem. Rev. 2023, 123 (13), 8488–8529. 10.1021/acs.chemrev.2c00914. 37279171 PMC10347433

[ref31] Eustis S.; El-Sayed M. A. Why Gold Nanoparticles Are More Precious than Pretty Gold: Noble Metal Surface Plasmon Resonance and Its Enhancement of the Radiative and Nonradiative Properties of Nanocrystals of Different Shapes. Chem. Soc. Rev. 2006, 35 (3), 209–217. 10.1039/B514191E. 16505915

[ref32] Chen H.; Shao L.; Ming T.; Sun Z.; Zhao C.; Yang B.; Wang J. Understanding the Photothermal Conversion Efficiency of Gold Nanocrystals. Small 2010, 6 (20), 2272–2280. 10.1002/smll.201001109. 20827680

[ref33] Radhasaran R.; Sathyan A.; Sivaraman R. K.; Sugumaran S.; Kamakshi K.; Sekhar K. C. Sensing, Antimicrobial and Photothermal Activity of Ultra-Stable Colloidal Copper Nanoparticles. Plasmonics 2022, 17 (6), 2521–2531. 10.1007/s11468-022-01742-4.

[ref34] Austin L. A.; Mackey M. A.; Dreaden E. C.; El-Sayed M. A. The Optical, Photothermal, and Facile Surface Chemical Properties of Gold and Silver Nanoparticles in Biodiagnostics, Therapy, and Drug Delivery. Arch. Toxicol. 2014, 88 (7), 1391–1417. 10.1007/s00204-014-1245-3. 24894431 PMC4136654

[ref35] Moya S. E.; Hernández R. R.; Angelomé P. C. Degradation of Mesoporous Silica Materials in Biological Milieu: The Gateway for Therapeutic Applications. Advanced NanoBiomed Research 2024, 4 (10), 2400005 10.1002/anbr.202400005.

[ref36] Liz-Marzán L. M.; Giersig M.; Mulvaney P. Synthesis of Nanosized Gold–Silica Core–Shell Particles. Langmuir 1996, 12 (18), 4329–4335. 10.1021/la9601871.

[ref37] Liu S.; Han M.-Y. Silica-Coated Metal Nanoparticles. Chem. Asian J. 2010, 5 (1), 36–45. 10.1002/asia.200900228. 19768718

[ref38] Wang F.; Cheng S.; Bao Z.; Wang J. Anisotropic Overgrowth of Metal Heterostructures Induced by a Site-Selective Silica Coating. Angew. Chem., Int. Ed. 2013, 52 (39), 10344–10348. 10.1002/anie.201304364. 23939636

[ref39] da Cruz Schneid A.; Albuquerque L. J. C.; Mondo G. B.; Ceolin M.; Picco A. S.; Cardoso M. B. Colloidal Stability and Degradability of Silica Nanoparticles in Biological Fluids: A Review. J. Sol-Gel Sci. Technol. 2022, 102 (1), 41–62. 10.1007/s10971-021-05695-8.

[ref40] Adleman J. R.; Boyd D. A.; Goodwin D. G.; Psaltis D. Heterogenous Catalysis Mediated by Plasmon Heating. Nano Lett. 2009, 9 (12), 4417–4423. 10.1021/nl902711n. 19908825

[ref41] Mascaretti L.; Schirato A.; Fornasiero P.; Boltasseva A.; Shalaev V. M.; Alabastri A.; Naldoni A. Challenges and Prospects of Plasmonic Metasurfaces for Photothermal Catalysis 2022, 11 (13), 3035–3056. 10.1515/nanoph-2022-0073. PMC11501173 39634672

[ref42] Li L.; Yang W.; Yang Q.; Guan Q.; Lu J.; Yu S.-H.; Jiang H.-L. Accelerating Chemo- and Regioselective Hydrogenation of Alkynes over Bimetallic Nanoparticles in a Metal–Organic Framework. ACS Catal. 2020, 10 (14), 7753–7762. 10.1021/acscatal.0c00177.

[ref43] Rashid U.; Bro-Jørgensen W.; Harilal K.; Sreelakshmi P.; Mondal R. R.; Chittari Pisharam V.; Parida K. N.; Geetharani K.; Hamill J. M.; Kaliginedi V. Chemistry of the Au–Thiol Interface through the Lens of Single-Molecule Flicker Noise Measurements. J. Am. Chem. Soc. 2024, 146 (13), 9063–9073. 10.1021/jacs.3c14079. 38381861 PMC10995995

[ref44] Pensa E.; Cortés E.; Corthey G.; Carro P.; Vericat C.; Fonticelli M. H.; Benítez G.; Rubert A. A.; Salvarezza R. C. The Chemistry of the Sulfur–Gold Interface: In Search of a Unified Model. Acc. Chem. Res. 2012, 45 (8), 1183–1192. 10.1021/ar200260p. 22444437

[ref45] Zhu X.; Jia H.; Zhu X.-M.; Cheng S.; Zhuo X.; Qin F.; Yang Z.; Wang J. Selective Pd Deposition on Au Nanobipyramids and Pd Site-Dependent Plasmonic Photocatalytic Activity. Adv. Funct. Mater. 2017, 27 (22), 1700016 10.1002/adfm.201700016.

[ref46] Bakhtiari A. B. S.; Hsiao D.; Jin G.; Gates B. D.; Branda N. R. An Efficient Method Based on the Photothermal Effect for the Release of Molecules from Metal Nanoparticle Surfaces. Angew. Chem., Int. Ed. 2009, 48 (23), 4166–4169. 10.1002/anie.200805303. 19408273

[ref47] Yang Q.; Xu Q.; Yu S.-H.; Jiang H.-L. Pd Nanocubes@ZIF-8: Integration of Plasmon-Driven Photothermal Conversion with a Metal–Organic Framework for Efficient and Selective Catalysis. Angew. Chem., Int. Ed. 2016, 55 (11), 3685–3689. 10.1002/anie.201510655. 26799948

[ref48] Trinh T. T.; Sato R.; Sakamoto M.; Fujiyoshi Y.; Haruta M.; Kurata H.; Teranishi T. Visible to Near-Infrared Plasmon-Enhanced Catalytic Activity of Pd Hexagonal Nanoplates for the Suzuki Coupling Reaction. Nanoscale 2015, 7 (29), 12435–12444. 10.1039/C5NR03841C. 26133744

[ref49] Huang X.; Li Y.; Chen Y.; Zhou H.; Duan X.; Huang Y. Plasmonic and Catalytic AuPd Nanowheels for the Efficient Conversion of Light into Chemical Energy. Angew. Chem., Int. Ed. 2013, 52 (23), 6063–6067. 10.1002/anie.201301096. 23616428

[ref50] Wang F.; Li C.; Chen H.; Jiang R.; Sun L.-D.; Li Q.; Wang J.; Yu J. C.; Yan C.-H. Plasmonic Harvesting of Light Energy for Suzuki Coupling Reactions. J. Am. Chem. Soc. 2013, 135 (15), 5588–5601. 10.1021/ja310501y. 23521598

[ref51] Matter M. E.; Tagnon C.; Stache E. E. Recent Applications of Photothermal Conversion in Organic Synthesis. ACS Cent. Sci. 2024, 10 (8), 1460–1472. 10.1021/acscentsci.4c00545. 39220710 PMC11363323

[ref52] Phillips S. J.; Ginder N. C.; Lear B. J. Rapid Photothermal Synthesis of Polyurethane from Blocked Isocyanates. Macromolecules 2022, 55 (16), 7232–7239. 10.1021/acs.macromol.2c00920.

[ref53] Haas K. M.; Lear B. J. Billion-Fold Rate Enhancement of Urethane Polymerization via the Photothermal Effect of Plasmonic Gold Nanoparticles. Chem. Sci. 2015, 6 (11), 6462–6467. 10.1039/C5SC02149A. 30090265 PMC6054102

[ref54] Asmussen S. V.; Arenas G. F.; Vallo C. I. Enhanced Degree of Polymerization of Methacrylate and Epoxy Resins by Plasmonic Heating of Embedded Silver Nanoparticles. Prog. Org. Coat. 2015, 88, 220–227. 10.1016/j.porgcoat.2015.06.032.

[ref55] Pastoriza-Santos I.; Kinnear C.; Pérez-Juste J.; Mulvaney P.; Liz-Marzán L. M. Plasmonic Polymer Nanocomposites. Nature Reviews Materials 2018, 3 (10), 375–391. 10.1038/s41578-018-0050-7.

[ref56] Fedoruk M.; Meixner M.; Carretero-Palacios S.; Lohmüller T.; Feldmann J. Nanolithography by Plasmonic Heating and Optical Manipulation of Gold Nanoparticles. ACS Nano 2013, 7 (9), 7648–7653. 10.1021/nn402124p. 23941522

[ref57] Boyd D. A.; Greengard L.; Brongersma M.; El-Naggar M. Y.; Goodwin D. G. Plasmon-Assisted Chemical Vapor Deposition. Nano Lett. 2006, 6 (11), 2592–2597. 10.1021/nl062061m. 17090097

[ref58] Kamarudheen R.; Kumari G.; Baldi A. Plasmon-Driven Synthesis of Individual Metal@semiconductor Core@shell Nanoparticles. Nat. Commun. 2020, 11 (1), 3957 10.1038/s41467-020-17789-y. 32770052 PMC7414885

[ref59] Cao L.; Barsic D. N.; Guichard A. R.; Brongersma M. L. Plasmon-Assisted Local Temperature Control to Pattern Individual Semiconductor Nanowires and Carbon Nanotubes. Nano Lett. 2007, 7 (11), 3523–3527. 10.1021/nl0722370. 17963415

[ref60] Robert H. M. L.; Kundrat F.; Bermúdez-Ureña E.; Rigneault H.; Monneret S.; Quidant R.; Polleux J.; Baffou G. Light-Assisted Solvothermal Chemistry Using Plasmonic Nanoparticles. ACS Omega 2016, 1 (1), 2–8. 10.1021/acsomega.6b00019. 31457112 PMC6640728

[ref61] Pandres E. P.; Crane M. J.; Davis E. J.; Pauzauskie P. J.; Holmberg V. C. Laser-Driven Growth of Semiconductor Nanowires from Colloidal Nanocrystals. ACS Nano 2021, 15 (5), 8653–8662. 10.1021/acsnano.1c00683. 33950682

[ref62] Thomas-Hillman I.; Laybourn A.; Dodds C.; Kingman S. W. Realising the Environmental Benefits of Metal–Organic Frameworks: Recent Advances in Microwave Synthesis. J. Mater. Chem. A 2018, 6 (25), 11564–11581. 10.1039/C8TA02919A.

[ref63] Głowniak S.; Szczęśniak B.; Choma J.; Jaroniec M. Recent Developments in Sonochemical Synthesis of Nanoporous Materials. Molecules 2023, 28 (6), 2639 10.3390/molecules28062639. 36985612 PMC10051140

[ref64] Carattino A.; Caldarola M.; Orrit M. Gold Nanoparticles as Absolute Nanothermometers. Nano Lett. 2018, 18 (2), 874–880. 10.1021/acs.nanolett.7b04145. 29272135 PMC5817619

[ref65] Jang D.; Yu S.; Chung K.; Yoo J.; Mota F. M.; Wang J.; Ahn D. J.; Kim S.; Kim D. H. Direct Deposition of Anatase TiO_2_ on Thermally Unstable Gold Nanobipyramid: Morphology-Conserved Plasmonic Nanohybrid for Combinational Photothermal and Photocatalytic Cancer Therapy. Applied Materials Today 2022, 27, 101472 10.1016/j.apmt.2022.101472.

[ref66] Kugelmass L. H.; Tagnon C.; Stache E. E. Photothermal Mediated Chemical Recycling to Monomers via Carbon Quantum Dots. J. Am. Chem. Soc. 2023, 145 (29), 16090–16097. 10.1021/jacs.3c04448. 37432654

